# *Arabidopsis* non-specific phospholipase C1: characterization and its involvement in response to heat stress

**DOI:** 10.3389/fpls.2015.00928

**Published:** 2015-11-04

**Authors:** Zuzana Krčková, Jitka Brouzdová, Michal Daněk, Daniela Kocourková, Dominique Rainteau, Eric Ruelland, Olga Valentová, Přemysl Pejchar, Jan Martinec

**Affiliations:** ^1^Institute of Experimental Botany, The Czech Academy of SciencesPrague, Czech Republic; ^2^Department of Biochemistry and Microbiology, University of Chemistry and Technology, PraguePrague, Czech Republic; ^3^1ERL Inserm U1157/UMR7203, Faculté de Medecine Pierre et Marie CurieParis, France; ^4^CNRS, UMR7618, Institut d’Ecologie et des Sciences de l’Environnement de ParisCréteil, France; ^5^Université Paris Est, Institut d’Ecologie et des Sciences de l’Environnement de Paris, UPECCréteil, France

**Keywords:** non-specific phospholipase C, heat stress, *Arabidopsis thaliana*, phospholipids, diacylglycerol

## Abstract

The *Arabidopsis* non-specific phospholipase C^1^ (NPC) protein family is encoded by the genes *NPC1 – NPC6*. It has been shown that NPC4 and NPC5 possess phospholipase C activity; NPC3 has lysophosphatidic acid phosphatase activity. NPC3, 4 and 5 play roles in the responses to hormones and abiotic stresses. NPC1, 2 and 6 has not been studied functionally yet. We found that *Arabidopsis* NPC1 expressed in *Escherichia coli* possesses phospholipase C activity *in vitro*. This protein was able to hydrolyse phosphatidylcholine to diacylglycerol. NPC1-green fluorescent protein was localized to secretory pathway compartments in *Arabidopsis* roots. In the knock out T-DNA insertion line NPC1 (*npc1*) basal thermotolerance was impaired compared with wild-type (WT); *npc1* exhibited significant decreases in survival rate and chlorophyll content at the seventh day after heat stress (HS). Conversely, plants overexpressing NPC1 (NPC1-OE) were more resistant to HS compared with WT. These findings suggest that NPC1 is involved in the plant response to heat.

## Introduction

Phospholipases are now recognized as key components of the phospholipid signaling network that regulates plant growth and development. This network includes, among other enzymes, PLC, PLD and phospholipases A_1_ and A_2_. These enzymes produce a set of second messenger molecules and lipid derivatives that are implicated in both plant metabolism and intracellular signaling. PLCs are able to cleave membrane phospholipids, which acilitates the release of water-soluble phosphorylated headgroups to form hydrophobic DAGs. PLCs can be generally divided into PI-PLC and PC-PLC according to substrate specificity ranges. PC-PLCs, which are also known in plants as NPC, are characterized by broader substrate ranges that include abundant PC (Nakamura et al., 2005; Pokotylo et al., 2013).

The *Arabidopsis* NPC gene family consists of six members, *NPC1–NPC6*. NPC4 was found to be a plasma membrane-bound protein (Nakamura et al., 2005; Pejchar et al., 2015). NPC5 is a cytosolic-localized enzyme expressed only in floral organs under normal conditions (Gaude et al., 2008; Pokotylo et al., 2013). NPC4 and NPC5 are able to hydrolyse PC; however, the hydrolytic activity of NPC5 is 40-fold lower than that of NPC4 (Gaude et al., 2008). Reddy et al. (2010) described NPC3 as an enzyme that has lysophosphatidic acid phosphatase activity instead of PLC activity. NPC1, NPC2, and NPC6, which have not been experimentally characterized yet, are supposed to contain putative N-terminal signal peptides and are predicted to localize to endomembranes and specific organelles (Pokotylo et al., 2013). For more detailed descriptions of the characteristics of NPCs, see Pokotylo et al. (2013) or Nakamura (2014).

Recent research has revealed important roles for NPCs as mediators of plant metabolism in relation to phospholipid-to-galactosyl DAG exchange (Andersson et al., 2005; Nakamura et al., 2005; Gaude et al., 2008), in growth and development related to hormone signaling (Peters et al., 2010; Wimalasekera et al., 2010) and in stress responses to changing environmental conditions (Scherer et al., 2002; Pejchar et al., 2010, 2015; Kocourková et al., 2011; Peters et al., 2014; Pejchar and Martinec, 2015).

One of the most studied stress conditions for plants is HS, due to its importance in agriculture. HS is mentioned in connection with global warming and changes in climatic conditions; HS is often accompanied by oxidative, osmotic and drought stress. As a defense response to HS, plants change membrane fluidity, cytoskeleton organization, protein conformation or enzymatic activities. Plants derive benefits from several specific proteins that help plants to get HS under control, such as HSFs (Liu et al., 2011), HSPs (Hong and Vierling, 2001) and small HSPs (Sun et al., 2002).

Hormones are also responsible for thermotolerance. Abscisic acid (ABA), SA, ethylene, zeatin, jasmonic acid and auxin have been implicated in the plant response to heat (Sakata et al., 2010; Qu et al., 2013; Dobrá et al., 2015). SA, ABA, and ethylene protect plants against heat-induced oxidative damage (Larkindale and Knight, 2002; Clarke et al., 2004). It was shown that oxidative damage already occurs after 1 h at 40°C (Larkindale and Knight, 2002). SA is not essential for acquired thermotolerance; however, this hormone influences basal thermotolerance (Clarke et al., 2004). Another important player in HS, nitric oxide, functions in HS signaling and stimulates the DNA-binding activity of HSFs and the accumulation of HSP18.2 through AtCaM3 (Xuan et al., 2010).

One of the basic thermotolerance mechanisms in plants is the alteration of the acyl composition of fatty acids. A *fad6* mutant that is deficient in chloroplast n-6 desaturase activity lacks unsaturated fatty acids and is more thermostable than the WT (Hugly et al., 1989). The trienoic acid in chloroplast lipids has a stronger effect on the thermotolerance of photosynthetic machinery than the trienoic fatty acid in non-chloroplast lipids (Murakami et al., 2000). The level of trienoic fatty acid (mainly 16:3) decreased in response to high temperature; the levels of 18:2 and 16:0 fatty acids increased. The time period of these changes in fatty acid composition corresponds with the time required for the new synthesis of fatty acids (Falcone et al., 2004).

Recent studies have demonstrated that HS response is also affected by phospholipases. Mishkind et al. (2009) reported that HS leads to dramatic increases in PIP_2_ and PA within 2 min after HS; these changes are mediated by PI phosphate kinase and PLD. Later, Zheng et al. (2012) showed that AtPLC9 (a member of the PI-PLC family in plants) is involved in the change in Ca^2+^ level induced by HS. AtPLC3, another member of the PI-PLC family, play similar role in HS. The effects of AtPLC3 and AtPLC9 are additive (Gao et al., 2014).

Here we show for the first time that *Arabidopsis* NPC1 possesses PC-hydrolysing PLC activity, is localized to the secretory pathway compartments, and is involved in the response to HS.

## Material and Methods

### Plant Material

*Arabidopsis thaliana* Columbia (Col-0) seeds were obtained from Lehle seeds (USA) and used as WT controls. Seeds of the T-DNA insertion line *npc1-1* SALK_027871 were obtained from NASC from the Salk Institute Genome Analysis Laboratory (SIGnAL) collection (Alonso, 2003). T-DNA insertion was confirmed by PCR using following primers: LBa1 TGGTTCACGTAGTGGGCCATCG, npc1R CAGAGACGGCCTCATAGTGAC and npc1L AGGGCACTGGTATGTGATTTG. PCR reactions were performed with PPP Master Mix (Top-Bio, Prague, Czech Republic).

Plants overexpressing *NPC1* and the fluorescent fusion protein NPC1:GFP were prepared as follows. NPC1 was amplified from *Arabidopsis* Col-0 cDNA using specific primers (forward 5′-CGAGTCGACAATGGCTTTCCGGCG-3′ and reverse 5′-TATGCGGCCGCTTGTAGCTTCCAATATACTTGTTGGTCC-3′), cloned into the pENTR3C entry vector (Invitrogen) and recombined by LR reaction into the Gateway binary vector pGWB2 (vector pGWB5 for NPC1:GFP) (Nakagawa et al., 2007) under the control of the CaMV 35S promoter. The construct was transferred into *Agrobacterium tumefaciens* strain GV2260 and used to transform *Arabidopsis* Col-0 WT plants by the floral dip method. Transformants were selected on agar plates containing 50 μg ml^-1^ kanamycin and 50 μg ml^-1^ hygromycin B. The expression level of *NPC1* in 10-day-old T2 seedlings of homozygous lines was measured using qRT-PCR. The line with the highest *NPC1* expression level was used in experiments.

#### Growth Conditions

Seeds were surface sterilized using a 30% (v/v) bleach solution for 10 min and rinsed five times with sterile water. The seedlings of different genotypes were grown on agar plates containing 2.2 g l^-1^ Murashige–Skoog basal salts and 0.8% (w/v) plant agar (Duchefa, pH 5.8) for horizontal position or 1% agar for vertical position. The plants were grown in a growth chamber at 22°C under long day conditions (16 h/8 h light/dark cycle) at 56 μmol m^-2^ s^-1^ light intensity.

For microscopy we used 5-day-old seedlings on 1% agar plates supplemented with 1% sucrose. For basal thermotolerance, lipid and hormone analysis we used 7-day-old seedlings on 0.8% agar plates, except measurement root length here we used 1% agar plates.

#### Transient Transformation of BY-2 Cells

The tobacco cell line BY-2 (*Nicotiana tabacum* L. cv Bright Yellow 2) was cultivated as described previously (Pejchar et al., 2010).

To obtain the 35S::NPC1:GFP construct for tobacco cell transformation, the coding sequence of NPC1 was obtained by PCR from *Arabidopsis* Col-0 cDNA using the specific primers 5′-CGTCTAGAATGGCTTTCCGGCGAG-3′ and 5′-CGCCCGGGGTAGCTTCCAATATACTTGTTGGTCCC-3′. The amplified fragment was then inserted into the *XbaI* and *SmaI* sites of a modified pGreenII binary vector (containing GFP between the *SalI* and *HindIII* restriction sites). The resulting construct was then isolated using Jet Star Plasmid Purification MIDI Kit (Genomed) and used for BY-2 particle bombardment transformation.

Two milliliters of 3-day-old culture of BY-2 cells were filtered and cells were retained on a filter paper disk (standard laboratory filtration paper, 80 g m^-2^). The filter disk was then placed onto a layer containing BY-2 medium supplemented with 0.7% agar in a 6 cm Petri dish. The Petri dishes were then placed in a PDS-1000 He biolistic device (Biorad). Particle bombardment was performed by applying 1100 psi. The coating of Au particles was performed as follows. Six micro liter of Au particles (1.6 μm in diameter, Biorad) in glycerol were mixed with 1 μg of pGreenII vector harboring 35S::NPC1:GFP, 6 μl CaCl_2_ (2.5 M) and 2.5 μl spermidine (0.1 M). The mixture was vortexed and subsequently centrifuged. The supernatant was removed, and the pellet was rinsed with 50 μl 70% ethanol. The pellet was vortexed and centrifuged again, and the supernatant was removed. The pellet was rinsed with 50 μl 100% ethanol, vortexed and spun, and the supernatant was removed. The resulting pellet was resuspended in 10 μl 100% ethanol and loaded onto the microcarrier membrane (Biorad). After bombardment, the plates were cultivated at 26.5°C in the dark. Cells were observed approximately 8 h after transformation.

### Expression and Purification of Recombinant NPC1 Protein

The cDNA for NPC1 (At1g07230) was amplified from *Arabidopsis* using specific primers (5′-GCCGTCGACCGATGATTGAATTCAAAAACTCC-3′ and 5′-CAGCGGCCGCTCAGTAGCTTCCAATATACTTGT-3′). The PCR product was digested with *NotI* and *SalI* and directly ligated in-frame into the expression vector pET-30a(+) (Novagen). The resulting plasmid containing the gene and a 6xHis tag was confirmed by sequencing and transformed into *Escherichia coli* strain BL21 by heat shock. Recombinant protein expression was induced by adding 0.1 mM IPTG when the A_600_ of the culture reached approximately 0.4. The induction continued overnight at 16 °C. The cells were harvested by centrifugation (5000 × *g*, 10 min), resuspended in lysis buffer (50 mM NaH_2_PO_4_, 300 mM NaCl and 10 mM imidazole) with Protease inhibitor cocktail tablets (Roche). Cells were sonicated after a 10 min treatment with lysozyme (1 mg ml^-1^). The lysed cell suspension was centrifuged (10000 × *g*, 10 min), and the supernatant was purified on Ni-NTA Agarose (Qiagen) according to the manufacturer’s instructions. The eluent was desalted on a PD-10 Desalting column (GE Healthcare) using desalting buffer (50 mM MES, 300 mM NaCl) and concentrated with Vivaspin ultrafiltration spin columns (SartoriusStedim). Purified protein was used for an enzyme activity assay.

Recombinant protein was separated under reducing conditions by 12% SDS-PAGE, followed by Coomassie blue staining or western blot. The 6xHis tag was detected by western blot analysis using an Anti-His HRP conjugate (Qiagen). HRP signal was visualized using SuperSignal West Pico chemiluminescent substrate (Thermo Scientific).

### Enzymatic Activity *In Vitro*

Expressed *Arabidopsis* NPC1 protein in *E. coli* were used to measure activity *in vitro*. Twenty micrograms of protein per assay was used. The protein concentration in the enzyme extracts was quantified using the method of Bradford (1976). Seventy five micro liter of proteins were mixed with 25 μl of substrate solution. The substrate solution consisted of 200 mM MES buffer (pH 6.5), 30.8 μM fluorescent PC (bodipy-PC, D-3771, Invitrogen, USA), 0.2 mM 1,2-dipalmitoyl-sn-glycerol-3-phosphocholine (Avanti Polar Lipids, USA) and 5.7 μM sodium deoxycholate (MP Biomedicals, USA). This substrate solution was gently shaken at 23°C for 30 min and then sonicated for 10 min. The reaction was initiated by mixing the substrate and protein solutions. The mixture was incubated at 28°C and 300 rpm for 2 h. The reaction was stopped by the addition of 400 μl of cold methanol/chloroform 2/1 (v/v). After 30 min, 200 μl KCl (0.1 M) was added. The lower phase (100 μl) was evaporated and re-dissolved in 50 μl of ethanol. Samples were applied to HP-TLC silica gel-60 plates (Merck KGaA, Darmstadt, Germany) by an ATS4 sampler (Camag, Muttenz, Switzerland). Plates were developed in a horizontal developing chamber (Camag) in a mobile phase of methanol/chloroform/water 25/65/4 (v/v/v). Plates were dried and labeled phospholipids were detected using a Fuji FLA-7000 fluorescence scanner (Fujifilm, Tokyo, Japan). The identification of the spot corresponding to bodipy-DAG was based on a comparison with a bodipy-DAG standard prepared as previously described (Pejchar et al., 2010).

### Microscopy

Stable *Arabidopsis* transformants expressing the fluorescent fusion protein NPC1:GFP under the control of the 35S promoter were used to investigate the subcellular localization of NPC1. Seedlings were observed using a Zeiss 510 DUO confocal laser-scanning microscope with a 40x C-Apochromat objective (WI, NA = 1.2) or Zeiss 880 confocal laser-scanning microscope with a 63x Plan-Apochromat objective (Oil, NA = 1.4). To counterstain the cell borders, propidium iodide (PI) staining was performed. Plants were immersed in 10 μg ml^-1^ PI solution for 40 s, rinsed three times in water and mounted in water on a microslide. Plasmolysis was induced by applying 1 M mannitol for 60 min. The seedlings were then stained with PI as described above and mounted in 1 M mannitol solution. FM4-64 was used to stain the plasma membrane and endosomes. Seedlings were immersed in 2 μM FM4-64 solution and placed on ice to prevent endocytosis for 5 min (Malínská et al., 2014). Seedlings were then mounted in water. For endosome trafficking FM4-64 labeling was performed at room temperature for 30 min. For endoplasmic reticulum staining 1 μM ER-Tracker^TM^ Blue-White DPX (Molecular Probes) was applied for 10 min.

GFP fluorescence was collected using 488 nm laser excitation and a 505-550 nm band pass emission filter. FM4-64, PI and mCherry fluorescence was collected using 561 nm laser excitation and a 575 nm (FM4-64 and mCherry) or a 650 nm (PI) long pass emission filter. ER-Tracker fluorescence was collected using 405 nm excitation and 410-498 band pass emission. Sequential scanning was performed to separate the signals. BY-2 cells were observed using a spinning disk confocal microscope (Yokogawa CSU-X1 on Nikon Ti-E platform) equipped with a 60x Plan Apochormat objective (WI, NA = 1.2) and Andor Zyla sCMOS camera. 488 and 561 nm laser excitation together with 525-030 nm and 607-036 nm single band filters (both Semrock Brightline) were used for fluorescence collection of GFP and FM4-64, respectively.

### Enzymatic Activity of NPC *In Situ*

Non-specific phospholipase C activity in BY-2 cells was measured as previously described (Pejchar et al., 2013) with some modification. Briefly, NPC activity was measured using bodipy-phosphatidylcholine (bodipy-PC, D-3771, Invitrogen, USA) as a substrate for a 6-day-old BY-2 cells. Cells were incubated with the substrate for 10 min. Half of the cells were transferred to 42°C, and the rest were kept at 20°C. The reaction was stopped with methanol/chloroform 2/1 (v/v) immediately after incubation (time 0 min) or after 5, 15, or 30 min of HS. Lipids were extracted and separated by HP-TLC, and bodipy-DAG was quantified.

### Basal Thermotolerance Analysis

To test survival rates, 22 seeds of WT and 22 seeds of mutants (*npc1-1* or NPC1-OE) were placed on each agar plate. To determine chlorophyll content, each half of plates contained 36 WT seedlings, 36 *npc1-1* seedlings and 36 NPC1-OE seedlings. One-week-old seedlings were transferred to 42°C for 45 min and thereafter to the normal growth conditions. Survival rates and chlorophyll contents were determined at 7 days after HS. Chlorophyll contents were measured according to Arnon (1949). To measure root length, 4-day-old seedlings of the same size were transferred onto agar plates (4 WT seedlings and 5 *npc1-1*/NPC1-OE seedlings). Seven-day-old plants were transferred to 42°C for 30 min. Length of root elongation after HS was measured after 14 days. Root measurements were performed using JMicroVision 1.2.7.

### Lipid Analyses

Lipids were extracted according to Welti et al. (2002). Briefly, 7-day-old *Arabidopsis* seedlings (WT, *npc1-1* and NPC1-OE genotypes) from control and HS (42°C, 45 min) plates were submerged in 3 ml 75°C preheated isopropanol with 0.01% BHT for 15 min. After the addition of 1.5 ml chloroform and 0.6 ml water, the samples were vortexed and agitated for 1 h at room temperature. Lipid extracts were transferred to glass tubes, and 4 ml chloroform/methanol 2/1 (v/v) with 0.01% BHT was added to the plant material; the samples were then shaken for 30 min. This step was repeated for three times. The extracts were washed with 1 ml 1 M KCl and 2 ml water and dried. Extracted tissues were dried at 105°C and weighed.

The patterns in the molecular species of the main phosphoglycerolipids were established by tandem mass spectrometry in multiple reaction mode (Rainteau et al., 2012). The HPLC separation of phosphoglycerolipid classes was performed using an Agilent 1100 HPLC system equipped with a 250 mm × 4 mm (length × internal diameter) 5 μm Lichrospher silica column. The mobile phases consisted of hexane/isopropanol/water 628/348/24 (v/v/v) supplemented with 10 mg l^-1^ ammonium formate and isopropanol/water 850/146 (v/v) supplemented with 10 mg l^-1^ ammonium formate. The gradient was the same as in Rainteau et al. (2012). The distinct glycerophospholipid classes were eluted successively as a function of the polar head group. Eluted lipids were continuously injected into a tandem mass spectrometer (QTrap2000, ABSciex). The MRM analysis was performed and acquired during the entire HPLC run (60 min).

### Hormone Analyses

7-day-old *Arabidopsis* WT, *npc1-1*, NPC1-OE seedlings were grown on agar plates and used for hormone analyses. The plants were kept in HS (42°C) or in control conditions for 45 min. After treatment, 150 mg of fresh weight was frozen in liquid nitrogen. The determination of plant hormones included extraction and purification on a mixed mode reversed phase – cation exchange SPE column (Oasis-MCX, Waters). The purified fractions were analyzed as in Dobrev and Vankova (2012) on an HPLC (Ultimate 3000, Dionex) coupled to a hybrid triple quadrupole/linear ion trap mass spectrometer (3200 Q TRAP, Applied Biosystems) set in selected reaction monitoring mode. Quantification of hormones was performed using an isotope dilution method with multilevel calibration curves (*r*^2^> 0.99). Data processing was carried out with Analyst 1.5 software (Applied Biosystems).

## Results

### AtNPC1 Encodes a Phospholipase C that Cleaves PC

**FIGURE 1:**
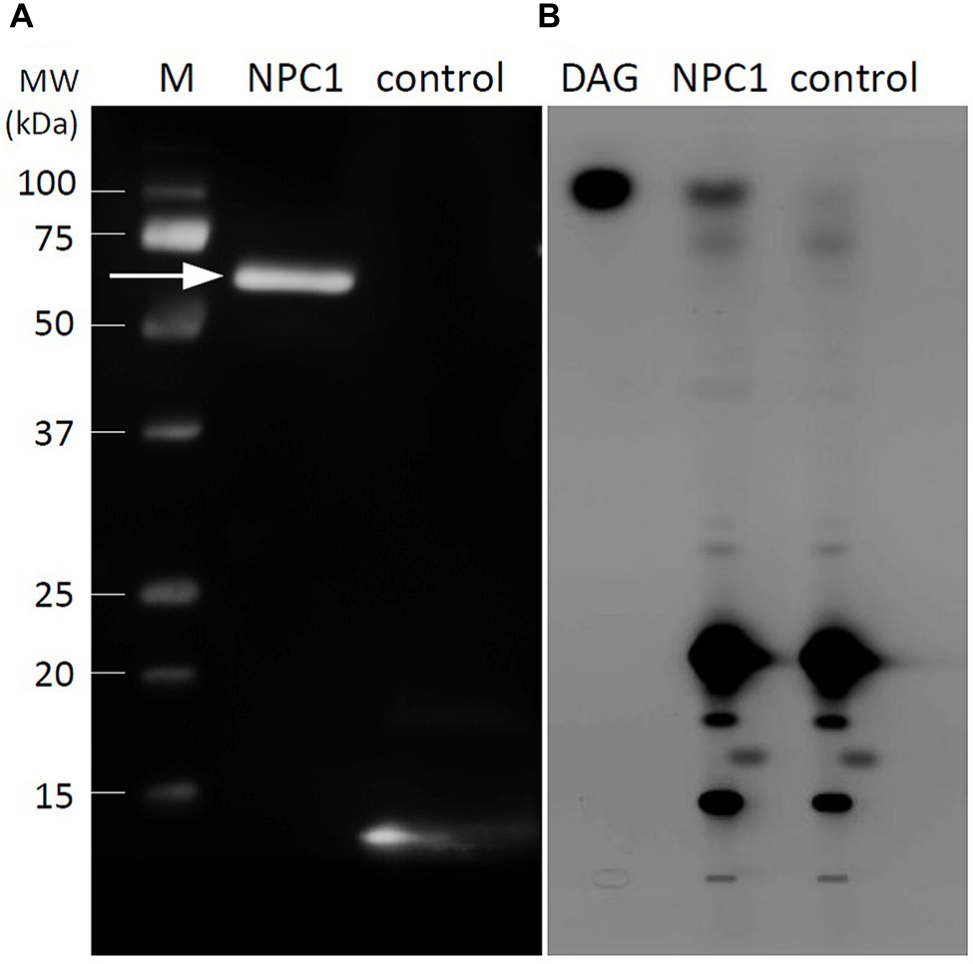
**Western blot analysis and *in vitro* activity of recombinant NPC1. (A)** Immunoblotting analysis of recombinant NPC1 protein after purification, desalting and concentration. The arrow indicates the 64 kDa protein corresponding to NPC1. The empty vector pET30 served as a control. Part of the expression region of the empty vector is transcribed with 6xHis and detected as a band of small size. **(B)** Activity assay of recombinant NPC1 protein and vector-only control performed with bodipy-PC as a substrate. Twenty micrograms of protein per assay was used. After the extraction of lipids and separation by high-performance thin layer chromatography, DAG was observed in NPC1. M, marker; DAG, diacylglycerol; NPC1, non-specific phospholipase C1.

It is believed that NPCs encode proteins with PLC activity. However, NPC3 shows lysophosphatidic acid phosphatase activity instead of PLC activity (Reddy et al., 2010). Therefore we studied the enzyme activity of recombinant NPC1. We prepared recombinant NPC1 by inserting AtNPC1 cDNA into the expression vector pET-30a(+)(pET30). This vector contains a 6xHis tag. The cleavage site between the signal peptide and the coding region was identified using SignalP 4.1 (Supplementary Figure S1A); the signal sequence was removed. The NPC1 produced in *E. coli* accumulated in inclusion bodies. To obtain greater solubility, we had to use a lower concentration of isopropyl β-thio-galactopyranoside (IPTG; 0.1 mM) and lower temperature (16°C) for a longer induction time. Protein was purified on Ni-NTA agarose, desalted to remove imidazole and concentrated. These conditions resulted in a sufficient amount of active enzyme with an expected size of 64 kDa. The prepared recombinant protein was detected on a western blot using an anti-His antibody. The empty vector pET30 served as a control (**Figure 1A**). Analyses of protein purity and purification steps are shown on Coomassie blue staining gel (Supplementary Figure S1B).

We measured the PC-hydrolysing activity of NPC1 *in vitro* with using a fluorescently labeled PC substrate (bodipy-PC). The reaction proceeded at 28°C for 2 h. Lipids were extracted and then separated by HP-TLC. The fluorescently labeled products were detected. The only spot that differed between NPC1 and the control line corresponded to DAG; this spot was therefore the product of NPC1 hydrolytic activity on PC (**Figure 1B**).

### NPC1 is Present in Secretory Pathway Compartments

**FIGURE 2:**
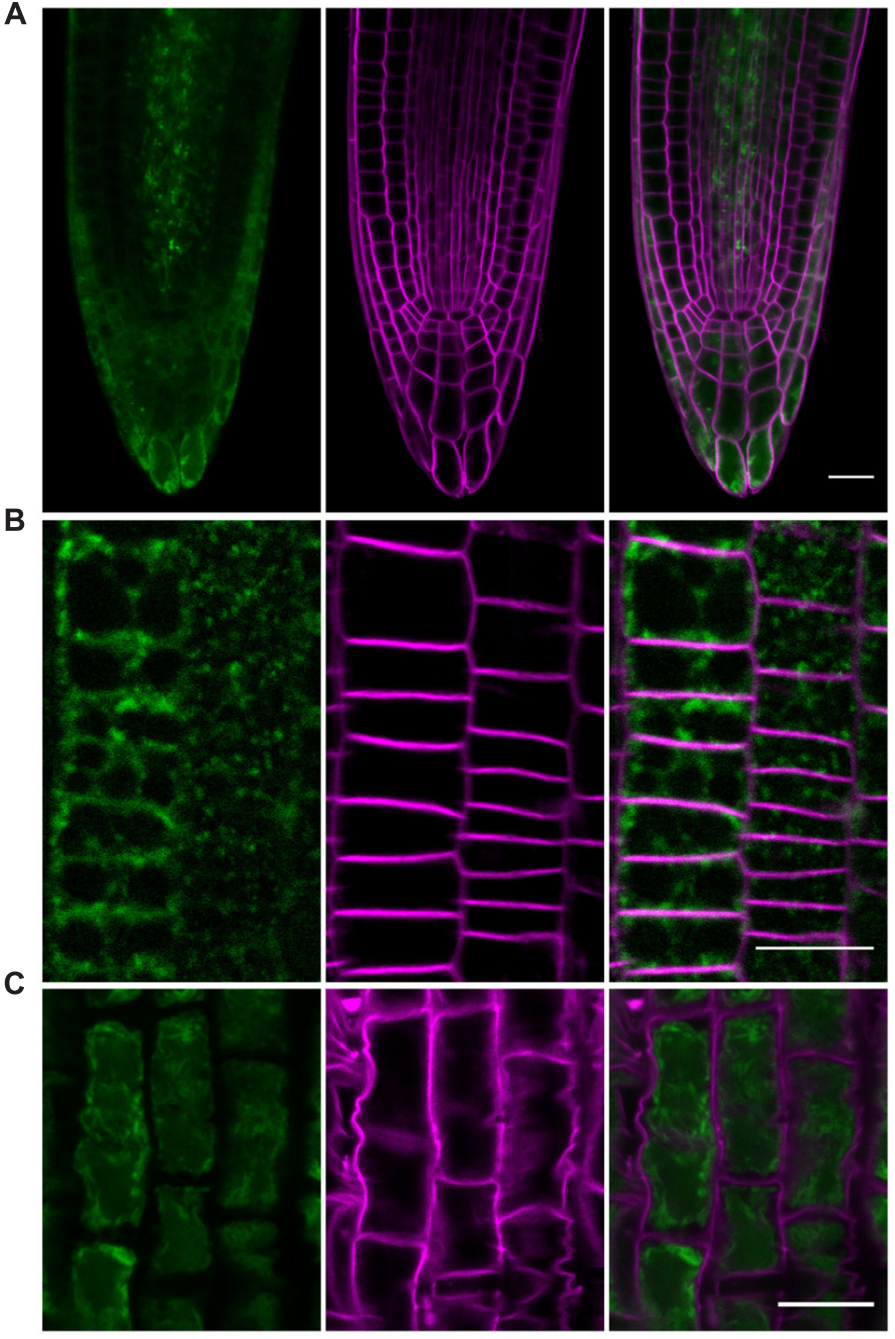
**Subcellular localization of NPC1:GFP in the roots of transgenic *Arabidopsis* seedlings.** Root elongation zone cells of 5-day-old seedlings of an *Arabidopsis* line expressing 35S::NPC1:GFP were observed. **(A)** NPC1:GFP is present in the cell interior and does not localize to the plasma membrane. The plasma membrane was stained with 2 μM FM4-64 for 5 min. NPC1:GFP signal (green, left) was detected in the cytoplasm; no co-localization with the FM4-64-stained plasma membrane (magenta, middle) was observed. **(B)** NPC1:GFP exhibits granular distribution in the cytoplasm. Roots were counterstained with FM4-64, as in **(A)**, to visualize cell borders/plasma membranes (magenta, middle). The NPC1:GFP signal (green, left) is not distributed evenly in the cytoplasm; it appears as highly pronounced in “dots” or granules within the cytoplasm. **(C)** NPC1:GFP is not localized to the cell walls. Plasmolysis was induced by the application of 1 M mannitol for 60 min. The cell walls were then stained with PI for 40 s. NPC1:GFP fluorescence (green, left) was observed only in the contracted protoplasts; no co-localization with PI-stained cell walls (magenta, middle) was observed. Bars represent 20 μm. NPC1, non-specific phospholipase C1; PI, propidium iodide.

The cellular localisation of protein of interest provides an opportunity to envisage its possible functional role. To determine the localization of the NPC1 protein, we prepared stable *Arabidopsis* transformants expressing a 35S::NPC1:GFP fusion protein and studied its localization in roots of 5-day-old seedlings using confocal microscopy. The GFP signal was localized to the cell interior (**Figure 2A**). FM4-64 labeling was used to counterstain cell borders. Detailed view of cortical cells revealed aggregates distributed within cytoplasm, mainly in the perinuclear region, as well as in the peripheral cytoplasm. No co-localization of GFP signal and FM4-64 labeled plasma membrane was observed (**Figure 2B**).

The presence of NPC1:GFP aggregates may suggest NPC1 association with internal membranes such as the endoplasmic reticulum or Golgi apparatus (GA) where its localization was predicted (Pokotylo et al., 2013). Such a protein could be eventually secreted to the extracellular space. As GFP signal was present in the cell periphery, we tested if GFP fluorescence was present in the cell wall by performing plasmolysis. We treated the roots with 1 M mannitol to induce pronounced plasmolysis. Cell walls were subsequently stained with PI. The GFP signal was observed only in contracted protoplasts. No signal was retained in the cell walls after plasmolysis (**Figure 2C**).

**FIGURE 3:**
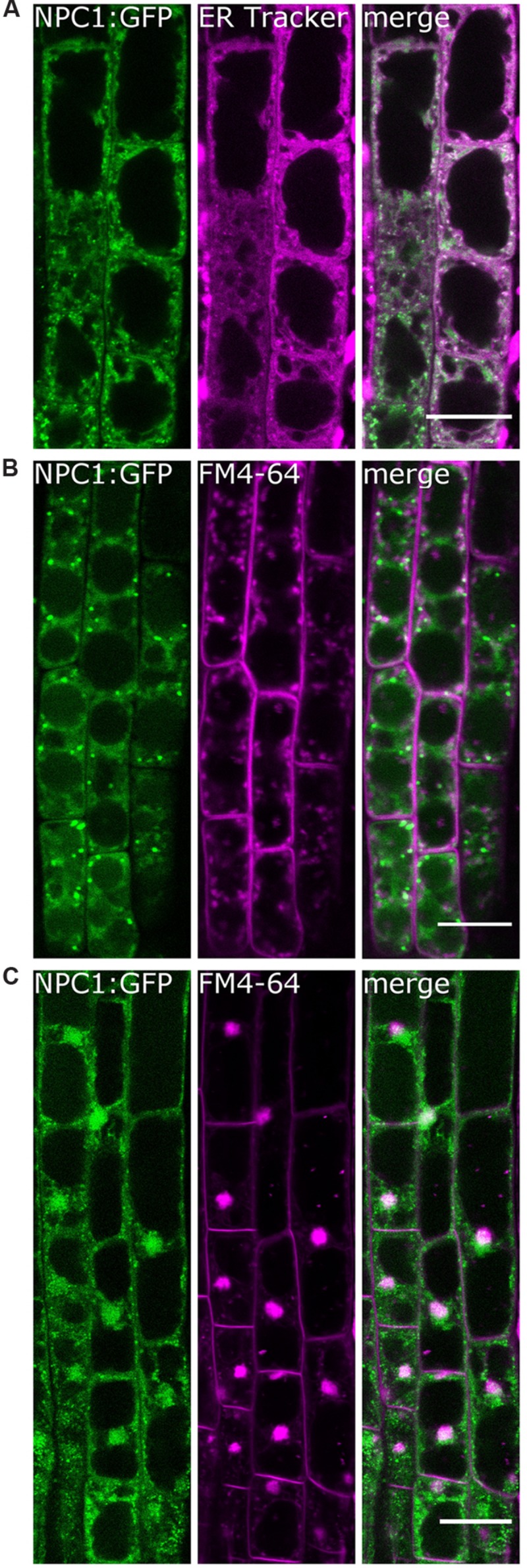
**NPC1:GFP is associated with secretory pathway compartments in root epidermal cells.** Root elongation zone cells of 5-day-old seedlings of an *Arabidopsis* line expressing 35S::NPC1:GFP were observed. **(A)** Roots were labeled with 1 μM ER-Tracker Blue White DPX (magenta) for 10 min. A partial colocalization with NPC1:GFP was found. **(B)** Roots were labeled with 2 μM FM4-64 (magenta) for 60 min. Only minor colocalization with NPC1:GFP was observed. **(C)** Roots were treated with 25 μM BFA and labeled as in **(B)**. Prominent BFA compartments were formed within which a high degree of colocalization of NPC1:GFP and FM4-64 was found. Bars represent 20 μm. BFA, brefeldin A; NPC1, non-specific phospholipase C1.

To further examine NPC1 localization in *Arabidopsis* roots, we used ER-Tracker Blue White DPX to specifically label endoplasmic reticulum (**Figure 3A**). A partial overlap between NPC1:GFP and ER-Tracker signal was observed suggesting an ER localization of at least subpopulation of NPC1. However, a part of NPC1:GFP signal was not colocalized with ER. To reveal whether this NPC1:GFP subpopulation was present at the GA, we labeled the roots with FM4-64, which is known to primarily label the plasma membrane and is used to track endosomes, the GA, prevacuolar compartments and the vacuole; the endoplasmic reticulum is not labeled with endocytosed FM4-64 (Bolte et al., 2004). Only minor co-localization of FM4-64-labeled internal membrane structures and NPC1:GFP puncta was observed (**Figure 3B**). We obtained similar results for BY-2 cell line transiently transformed with 35S::NPC1:GFP by particle bombardment method (Supplementary Figure S2). In order to identify the localization of remaining NPC1:GFP signal we treated the roots with BFA together with FM4-64. BFA induces aggregation of endosomal, GA and trans-Golgi network (TGN)-derived vesicles to form so called BFA compartments or BFA aggregates that colocalize with endocyted FM4-64 (Geldner et al., 2003, 2009; Grebe et al., 2003; Dettmer et al., 2006). BFA treatment induced a massive aggregation of NPC1:GFP positive structures that profoundly colocalized with FM4-64 labeled compartments (**Figure 3C**). Based on these findings we propose that, in addition to the ER localization, NPC1:GFP is present at GA and/or TGN as well.

### *In Situ* NPC Activity Increases During Heat Stress

**FIGURE 4:**
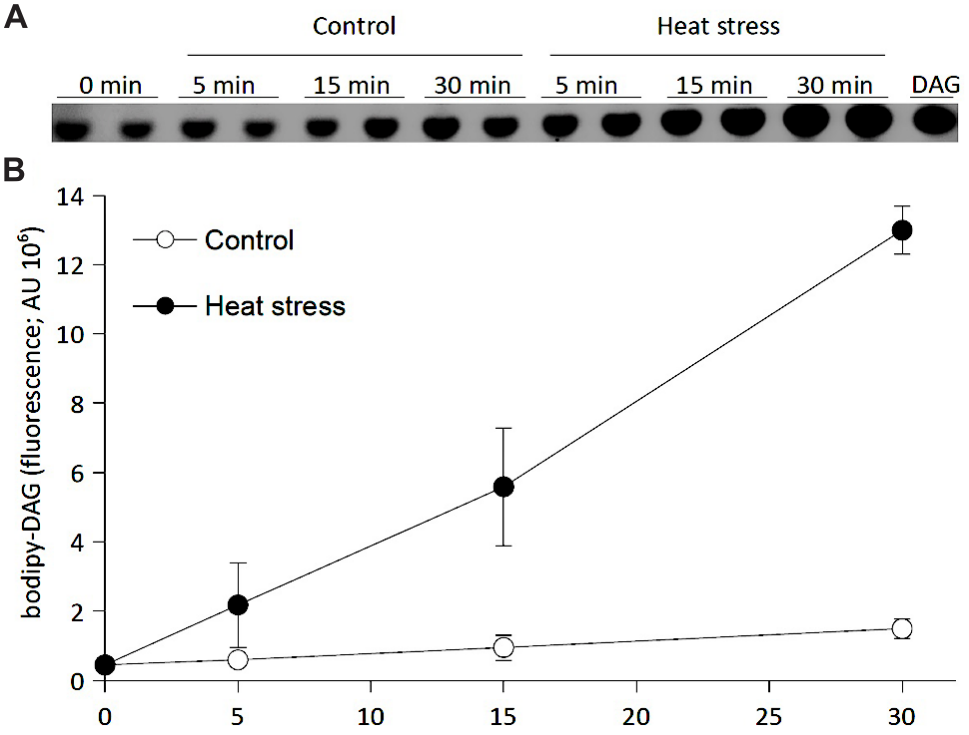
**Effect of heat stress on NPC activity in BY-2 tobacco cells.** Six-day-old BY-2 cells were incubated with bodipy-PC as a substrate for 10 min. Half of the cells were transferred to 42°C, and the rest were kept at 20°C. The reaction was stopped at the times indicated. Lipids were extracted and separated by HP-TLC, and bodipy-DAG fluorescence was quantified. **(A)** HP-TLC chromatogram of bodipy-DAG, the product of NPC activity. **(B)** The quantification of bodipy-DAG fluorescence. Data represent means ± SD from independently analyzed parallel samples. This experiment was repeated twice with similar results. DAG, diacylglycerol; HP-TLC, high-performance thin layer chromatography; NPC, non-specific phospholipase C; PC, phosphatidylcholine.

Several studies revealed that phospholipases, namely PI-PLC and PLD, play a role in HS (Mishkind et al., 2009; Zheng et al., 2012; Gao et al., 2014). To test wheteher NPC1 may be also involved in HS, we first measured NPC activity in BY-2 cells using a fluorescently labelled substrate (Pejchar et al., 2010, 2013) to determine whether activity changes after HS. We incubated bodipy-PC with BY-2 cells for 10 min. We then transferred half of the cells to 42°C; the remaining cells were kept under control conditions (20°C). The reaction was stopped immediately (0 min) or after 5, 15, or 30 min. Lipids were extracted and separated by HP-TLC. Spots corresponding to bodipy-DAG were quantified (**Figure 4**). The level of bodipy-DAG in this experimental setup corresponds mainly to NPC activity (Pejchar et al., 2010). However, the assay cannot fully exclude the possibility that PLD/PAP pathway is partially involved. We found increasing, time-dependent NPC activity during HS. The NPC activity increased fourfold in stressed cells compared with the control after 5 min; NPC activity was six- and ninefold greater after 15 and 30 min, respectively (**Figure 4**). We also measured activity during recovery after HS. The cells were stressed for 30 min and then returned to control conditions. Activity was measured after 15 min, 1, 3, 5, and 24 h of recovery. We observed that the activity remained higher in stressed cells in the first hour after HS. Later, the NPC activity in stressed cells returned to the level of control cells (data not shown). Taken together, these results suggest that NPC activity increased rapidly during HS and stays high during the short, transient period of recovery after HS in BY-2 cells.

### AtNPC1 is Involved in Basal Thermotolerance

Given that NPC activity was altered during HS, we stressed *Arabidopsis* NPC1 T-DNA insertion mutant (*npc1-1*) to monitor the following three different phenotypic characteristics: survival rate, chlorophyll content and root length. We cultivated 7-day-old *Arabidopsis* seedlings at 42°C for 45 min (30 min for root length measurements) and compared the phenotype of stressed mutant and WT after 7 days (2 weeks for root length measurements).

**FIGURE 5:**
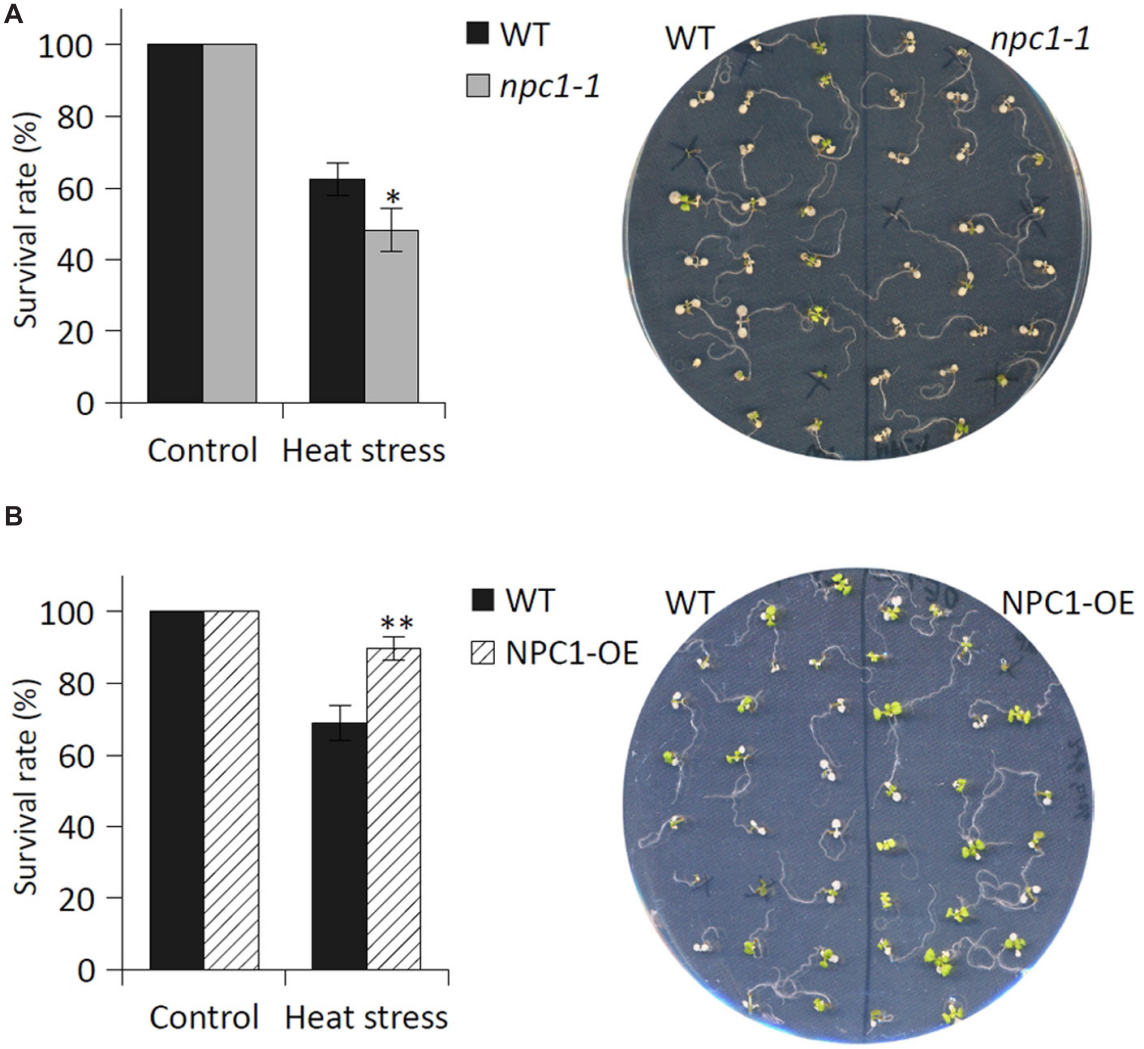
**The effect of HS on the survival rate of *Arabidopsis* WT, *npc1-1* and NPC1-overexpressing seedlings.** Seven-day-old *Arabidopsis* **(A)** WT and *npc1-1* seedlings or **(B)** WT and NPC1-OE seedlings were grown on agar plates (22 seedlings of each genotype on one plate) at 22°C. The plates were exposed to HS (42°C, 45 min), then returned to the control conditions. The survival rate was determined 7 days after HS. Each value is the mean ± SE. Asterisks denote significant differences compared with the WT (two-tailed Student‘s test; *n* = nine plates for *npc1-1* and *n* = six plates for NPC1-OE; ^∗^*P* < 0.05; ^∗∗^*P* < 0.01). This experiment was repeated four times (for *npc1-1* seedlings) or twice (for NPC1-OE seedlings) with similar results.

**FIGURE 6:**
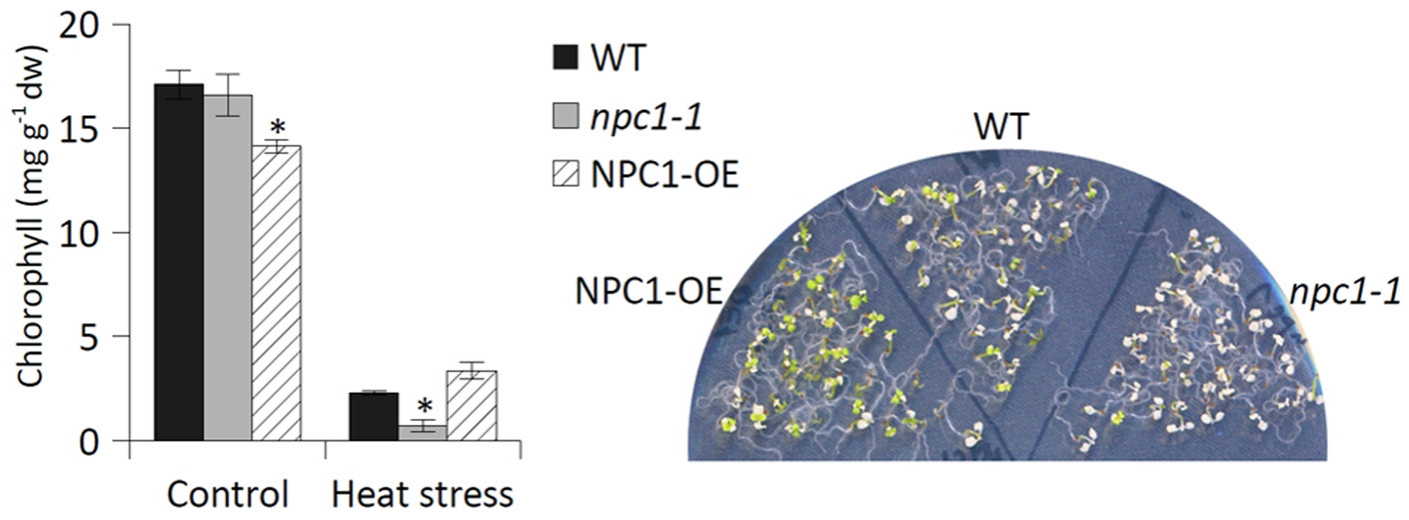
**The effect of HS on the chlorophyll content of *Arabidopsis* WT, *npc1-1* and NPC1-overexpressing seedlings.** Seven-day-old *Arabidopsis* seedlings were grown on agar plates (36 seedlings of each genotype on one plate) at 22°C. The plates were exposed to HS (42°C, 45 min), then returned to the control conditions. The chlorophyll content was determined 7 days after HS. Each value is the mean ± SE. Asterisks denote significant differences compared with the WT (two-tailed Student‘s test; *n* = 3 plates; ^∗^*P* < 0.05). This experiment was repeated twice with similar results. dw; dry weight.

**FIGURE 7:**
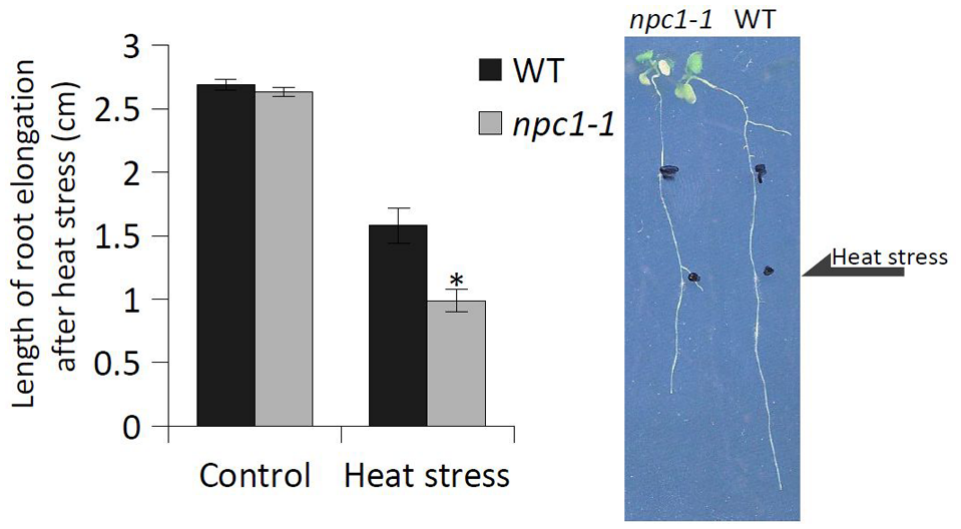
**The effect of HS on the root length of *Arabidopsis* WT and *npc1-1* seedlings.** Seven-day-old *Arabidopsis* seedlings were grown on agar plates (four seedlings of WT and five seedlings of *npc1-1* on one plate) at 22°C. The plates were exposed to HS (42°C, 30 min), then returned to the control conditions. Root elongation was determined 14 days after HS. Each value is the mean ± SE. Asterisk denotes significant difference compared with the WT (two-tailed Student’s test; *n* = six plates; ^∗^*P* < 0.05). This experiment was repeated three times with similar results.

We found that the survival rate of *npc1-1* (for mutant characterization, see Supplementary Figure S3) was significantly lower after HS (48% of control) compared with WT (62% of control) (**Figure 5A**). To confirm the possible involvement of NPC1 in HS, we prepared a stable *Arabidopsis* line overexpressing the NPC1 gene (NPC1-OE) (Supplementary Figure S3). In contrast to *npc1-1*, we observed an increase in the survival rate for NPC1-OE after HS (85% of control) compared with WT (68% of control) (**Figure 5B**). For both mutant lines, no differences were found under normal conditions (**Figure 5**, Supplementary Figure S4). Next, we analyzed the chlorophyll content of WT, *npc1-1* and NPC1-OE under normal and HS conditions (**Figure 6**). We detected no variations between WT and *npc1-1* while NPC1-OE seedlings contained less chlorophyll compared with WT under normal conditions. HS caused a dramatic decrease in chlorophyll content in all lines tested. More importantly, the ratios of chlorophyll content in HS/normal conditions were different in all lines; these ratios reached 13, 4, and 26% in WT, *npc1-1* and NPC1-OE seedlings, respectively. We detected no difference in root length between WT and *npc1-1* under normal conditions (**Figure 7**) and between WT and NPC1-OE under HS and normal conditions (data not shown). However, a significant reduction in length of root elongation was observed in *npc1-1* after HS (60% of that observed in heat stressed WT plants) (**Figure 7**). It should be noted that the time of HS treatment was reduced from 45 to 30 min when analyzing the length of root elongation after HS; all root growth stopped after longer HS treatment. Taken together, our results showed that *npc1-1* is more sensitive to HS and NPC1-OE has increased resistance to HS compared with WT.

### The Alterations of NPC1 Level has No Impact on ROS Accumulation or Transcript Level of *HSP18.2, HSP70, MBF1c* and *DREB2A*

To understand the molecular mechanism by which NPC1 is involved in thermotolerance, we first focused on HS signaling; NPC1 may serve as a signaling protein. We measured the accumulation of ROS and the expression of HSPs as early and late heat signaling markers.

Reactions to stress are mostly coupled with the accumulation of ROS. A relationship also exists between ROS and the HSR (Ruelland and Zachowski, 2010; Wang et al., 2014). We assessed ROS accumulation using 2′, 7′-dichlorofluorescein diacetate as a ROS detection reagent according to Wang et al. (2014) after 30 min periods of HS (45°C). No difference in ROS accumulation pattern among WT, *npc1-1* and NPC1-OE seedlings was observed (data not shown).

The synthesis of HSPs is evident at the end of the HS signaling chain (Larkindale et al., 2005). Given that the level of HSP expression is used as a marker of alteration in HS signaling, we measured the expression of *HSP18.2* and *HSP70*. We also measured the expression of *MBF1c*, a transcriptional regulator required for basal thermotolerance (Suzuki et al., 2011), and *DREB2A*, one of the key transcription factors of the HSR in plants (Schramm et al., 2008). Seven-day-old *Arabidopsis* seedlings were heated (37°C) for 0.5, 1, and 3 h, and levels of transcripts were determined by qRT-PCR according to Kocourková et al. (2011). No differences in the expression of any of the genes tested were observed between WT, *npc1-1* and NPC1-OE seedlings (data not shown).

### Alteration of Basal Thermotolerance in NPC1 Mutants is Not Caused by Rapid Changes in Lipid or Hormone Levels

Temperature stress, including HS, is likely to be perceived through changes in membrane fluidity (Ruelland and Zachowski, 2010). Membrane fluidity can be altered according to the composition of lipids. Lipid unsaturation is associated with more fluid membranes (Vaultier et al., 2006). Changes in membrane composition affects heat sensing (Saidi et al., 2010) and the adaptation to HS. It is possible that the manipulation of NPC1 transcripts has an impact on the lipid composition of membranes and, subsequently, the thermotolerance of the studied NPC1 genotypes.

The composition of major phosphoglycerolipids was analyzed by mass spectrometry in multiple reaction mode. This type of analysis provides access to the molecular species of lipids that is coupled to the fatty acids esterified on the *sn*-1 and *sn*-2 of their glycerol backbone.

The pattern of molecular species was first analyzed for lipids extracted from non-stressed WT, *npc1-1* or NPC1-OE seedlings (Supplementary Figure S5). No difference between the three types of plants could be detected for any of the analyzed phosphoglycerolipids.

The molecular species composition of lipids extracted from WT plants that were submitted to a 45 min HS (42°C) was also analyzed. After HS, plants were returned to control temperature. Lipids were extracted at either 2 or 24 h (Supplementary Figure S6) after the return to control temperature. For PE, we see no difference between treated plants and control plants. For PG, the only significant change detected concerns a very minor species; 18:2/18:1-PG decreases in lipids extracted from plants returned to control temperature for 2 h compared with control plants. This decrease is not detected after 24 h of recuperation. Major changes are associated with PC and PA. We see a decrease in 16:0/18:2- and 18:2/18:2- associated with an increase in 18:3/18:3- PC. If we consider the lipids extracted at 24 h after the return to control temperature, the decrease in 16:0/18:2- and 18:2/18:2- and the increase in 18:3/18:3-PC is even more pronounced. As for PA, a decrease in 16:0/18:2 and an increase in 18:1/18:3-PA are observed in plants submitted to heat treatment followed by 2 h of recuperation compared with control plants. These changes are still detected in PA extracted 24 h after the return to control temperature. This treatment is also associated with a decrease in 18:2/18:2- and an increase in 18:3/18:3-PA.

The lipids were also extracted in a heat stressed mutant and overexpressing lines. No differences in lipid composition were observed between WT, *npc1-1* and NPC1-OE plants (data not shown). In particular, the decrease in 16:0/18:2- and 18:2/18:2-PC and increase in 18:3/18:3-PC were the same in the three genotypes. Besides, a principal component analysis was run with the compositions of PCs from plants submitted to heat and back to control temperatures for 2 or 24 h and their respective controls (Supplementary Figure S7). Data are clustered by the treatments, i.e., PCs from plants submitted to 45 min HS (lipids extracted 24 h after plants were back at normal temperature) are together whatever the genotypes; another cluster is PCs from plants submitted to 45 min HS (lipids extracted 2 h after plants were placed back at normal temperature). The controls are together, with a difference between 24 h control and 2 h control. The main differentiating vector is F1 axis, with 18:3/18:3, 18:2/18:2 and 16:0/18:2 contributing the most to this axis, with HS plants (lipids extracted 24 h after plants were back at normal temperature) colocalising with 18:3/18:3. This is in accordance with the result description above.

Abscisic acid, SA, ethylene, zeatin, jasmonic acid and auxin have been implicated in the plant response to heat (Larkindale and Huang, 2005; Clarke et al., 2009; Sakata et al., 2010; Qu et al., 2013; Dobrá et al., 2015). Therefore, we determined the levels of hormones in control and heat stressed (42°C, 45 min) 7-day-old WT, *npc1-1* and NPC1-OE seedlings to examine whether hormone alteration is the basis of altered heat sensitivity in *NPC1* mutants.

Among these hormones, only the endogenous levels of ABA, its catabolite dihydrophaseic acid and certain cytokinines (trans-zeatin-9-glucoside, isopentenyl adenosine, isopentenyl adenine-7-glucoside and isopentenyl adenosine monophosphate) decreased significantly after 45 min of HS (42°C) (Supplementary Figure S8). There were no significant differences in the hormone levels between WT and mutant plants under the control conditions as well as after HS (data not shown).

## Discussion

NPC1 belongs to NPC protein family. NPC4 and NPC5 are members of this family that show PLC activity with specificity for major membrane phospholipids, such as PC or PE. Recombinant NPC4, when expressed in *E. coli*, showed specific activity toward PC and PE; this activity was not calcium dependent. For PA and PIP_2_, no activity was detected (Nakamura et al., 2005). NPC5 was also heterologously expressed in *E. coli*; this protein was able to cleave PC and PE to produce DAG. Curiously, heterologously expressed AtNPC3 has lysophosphatidic acid phosphatase activity (Reddy et al., 2010). Therefore, we expressed NPC1 protein in *E. coli.* Out of the six *Arabidopsis* NPCs, NPC1, NPC2, and NPC6 have putative transit peptides at the N-terminus that are predicted to be signal peptides (Nakamura et al., 2005; Pokotylo et al., 2013). According to Tan et al. (1997), we decided to remove the signal peptide sequence during the preparation of recombinant protein. *E. coli* may lack the appropriate protease to convert the pre-protein to the active protein. An enzyme assay with recombinant NPC1 shows PLC activity toward PC to produce DAG. Substrate specificity and the biochemical characteristics of NPC1 remain unclear and require further investigation.

NPC1 was predicted to localize to the endoplasmic reticulum based on the presence of a signal sequence (Pokotylo et al., 2013). However, localization in *Arabidopsis* plants stably overexpressing NPC1:GFP was not unambiguous. In root cells, NPC1:GFP was present in the cytoplasm and formed dots or puncta with pronounced fluorescence. A significant part of the GFP signal was found to colocalize with ER. Minor colocalization with endocyted FM4-64 was observed. The degree of colocalization dramatically increased in case of BFA-treated roots where pronounced colocalization of FM4-64 and NPC1:GFP was determined within BFA-induced compartments. In plant cells, BFA-induced compartments recruiting endocyted FM4-64 represent TGN whereas GA vesicles aggregating upon BFA-treatment were found not to colocalize with endocyted FM4-64 (Lam et al., 2009). Moreover, we found very similar localization pattern in BY-2 cells transiently transformed with NPC1:GFP, i.e., principal localization at ER-resembling structures and minor colocalization with FM4-64 labeled endosomes. Hence we suggest that NPC1 is present predominantly at ER and TGN with minor occurrence at GA or may be accumulated at TGN proceeding through the secretory pathway beginning at ER. However, it is necessary to add that using 35S-driven overexpression lines might have possibly affected localization pattern of NPC1. Moreover, results obtained using relatively high dose BFA treatment cannot point to exact molecular function of NPC1. Regardless, observed NPC1 localization seems rather different from that of *Arabidopsis* NPC4 and NPC5 which were found to localize mainly to plasma membrane (Nakamura et al., 2005) and cytosolic fraction (Gaude et al., 2008), respectively. On the other hand, minor amounts of NPC5 and NPC4 were found in microsomal fraction (Gaude et al., 2008) and ER/GA (Nakamura et al., 2005) respectively. All the three *Arabidopsis* NPC isoforms thus exhibit – to various extent – membrane association localization despite the lack of a clear transmembrane region (Nakamura et al., 2005). The rice homologue of NPC1 was expressed in *Nicotiana benthamiana* and localized to the cytoplasm and the nucleus, forming dot-like structures in cytoplasm that were eventually found to co-localize with chlorophyll autofluorescence (Singh et al., 2013). We were not able to detect GFP signals in the leaves; however, the dots or puncta found in our study are far smaller (under 1 μm^2^, data not shown) than the published size range of *Arabidopsis* root plastids (Kojo et al., 2009).

It has been documented that phospholipases are involved in the plant response to HS (Horváth et al., 2012; Higashi et al., 2015). Mishkind et al. (2009) reported on the rapid accumulation of PA and PIP_2_ within a sudden increase in temperature. Similarly, we detected a rapid increase in the level of labeled DAG. We have shown previously (Pejchar et al., 2010) that the level of labeled DAG corresponds with NPC activity. The homozygous *NPC1* knockout mutant showed increased sensitivity to HS compared with WT. Opposite behavior was shown by an *NPC1*-overexpressing line. Thermotolerance in this line was significantly higher than that in WT plants. Based on these results, it is possible to conclude that NPC1 is involved in thermotolerance in *Arabidopsis*.

The composition of membrane lipids plays a prominent role in the sensitivity of plants to temperature changes. We could not *a priori* rule out that manipulation of NPC protein levels impacts the lipid composition of membranes. Possibly the altered lipid composition of membranes could result in differences in responses to HS in different plant genotypes. The molecular patterns of the major phosphoglycerolipids were established, and no differences between genotypes were revealed. Therefore, the observed differences in heat responses in the three genotypes most likely did not result from differences in heat perception. Interestingly, heat treatment was not associated with major changes in the composition of molecular lipid species. Major changes were observed only in PC and PA; an increase in 18:3/18:3- species and decreases in 16:0/18:2- and 18:2/18:2- species occurred. Cultivation at elevated temperatures is usually associated with an increase in the saturation of fatty acids and a decrease in 18:3 fatty acid content (Falcone et al., 2004). This discrepancy is apparent. In our case, the HS is acute and short. In Falcone et al. (2004), an increase in 16:0 species and a decrease in 18:3-fatty acids were detectable no sooner than 100 h after transfer to elevated growth temperatures (29°C). In our experiment, the lipids are extracted from plants that were returned to the control temperature for 2 or 24 h after 45 min of acute HS. Plants not only experience heat, they experience acute heat and a temperature drop. Clearly, the differences in lipid composition between plants exposed to a continuous but mild HS versus plants exposed to an acute but short HS followed by a return to control temperatures is worth more investigation.

Changes in endogenous hormone levels in HS conditions have been reported; in addition, hormone mutants have shown altered heat sensitivity (Wahid et al., 2007; Dobrá et al., 2015). Involvement of some members of NPC family in hormone signaling was observed. Positive modulation of ABA response to salt stress with NPC4 was demonstrated (Peters et al., 2010; Wimalasekera et al., 2010; Kocourková et al., 2011). Moreover, in auxin and brassinolide-treated *P_NPC3_:GUS* and *P_NPC4_:GUS* seedlings, increase of GUS activity was visible. *P_NPC4_:GUS* seedlings also responded to cytokinine with increased GUS activity in young leaves (Wimalasekera et al., 2010). However, no significant differences between WT and *npc1-1* plants in either control or HS conditions were observed. It should be noted that we focused on differences that were observable before and immediately after HS. Differences at a later recovery period might be important as well. For example, Larkindale and Huang (2005) detected the accumulation of ABA and ethylene during a stress recovery period. Therefore, a more in-depth investigation of the phenomenon may be required to rule out NPC1–hormone interactions during HS.

The mechanism of involvement of PI-PLC and PLD in HS was studied. It was shown (Zheng et al., 2012; Gao et al., 2014) that AtPI-PLC3 and AtPI-PLC9 are involved in HS signal transduction via inositol 1,4,5-trisphosphate, which triggers changes in calcium ion levels. This change results in the altered expression of HSPs. The role of PLD in HS is far less clear. PA, the enzymatic product of PLD is presumable key molecule of action. It was documented that PA regulates ROS production (Park et al., 2004). Another possibility of PA involvement in HS is via PA-interacting protein complexes. Testerink et al. (2004) identified HSP 90 as a PA-binding target in plants. NPCs cleave ordinary membrane phospholipids, such as PC or PE, and release DAG. DAG may be quickly converted by DAG kinase to PA (Meijer and Munnik, 2003). An example of such mechanism is involvement of NPC4 in salt and drought stress response. Peters et al. (2010) showed that modulation of ABA response to salt and drought stress functions via PA and that the PA is product of NPC4-produced DAG conversion with DAG kinase. However, a number of studies imply that DAG may function as signaling molecule (Dong et al., 2012). To reveal whether NPC1 functions as a signaling protein in HS, we measured changes in ROS pattern and the expression of several heat marker genes in *npc1-1*, NPC1-OE and WT plants. Neither ROS pattern nor expression of heat marker genes *HSP18.2, HSP70, DREB2A* or *MBF1c* differ among inspected mutants. Based on these results it is possible to hypothesize that NPC1 is not likely to be involved in HS signaling or rapid alterations in hormone and lipid levels.

## Conflict of Interest Statement

The authors declare that the research was conducted in the absence of any commercial or financial relationships that could be construed as a potential conflict of interest.

## Abbreviations

BFA: brefeldin A
BHT: butylated hydroxytoluene
DAG: diacylglycerol
HP-TLC: high-performance thin layer chromatography
HRP: horseradish peroxidase
HS: heat stress
HSF: heat shock transcription factor
HSP: heat shock protein
HSR: heat stress response
IPTG: isopropyl *β*-thio-galactopyranoside
NPC: non-specific phospholipase C
PA: phosphatidic acid
PC: phosphatidylcholine
PC-PLC: phosphatidylcholine-specific phospholipases C
PE: phosphatidylethanolamine
PG: phosphatidylglycerol
PI: phosphatidylinositol
PIP_2_: phosphatidylinositol 4,5-bisphosphate
PI-PLC: phosphatidylinositol-specific phospholipases C
PLC: phospholipase C
PLD: phospholipase D
qRT-PCR: quantitative real time PCR
ROS: reactive oxygen species
SA: salicylic acid
WT: wild-type
