# Supplementary material for: Arabidopsis non-specific phospholipase C1: characterization and its involvement in response to heat stress
Source: Front Plant Sci. 2015 Nov 4;6:928. doi: 10.3389/fpls.2015.00928 (PMC4631941; doi:10.3389/fpls.2015.00928)
Supplement: Supplementary file 1 [file Image_1.PDF]

SUPPORTING INFORMATION

Figure S1. Prediction of a signal peptide cleavage site and analyses of purification of the recombinant NPC1

Figure S2. NPC1:GFP localization in tobacco BY-2 cells

Figure S3. Characterisation of *npc1-1* and NPC1-OE mutant lines

Figure S4. Survival rate of *Arabidopsis thaliana* WT, *npc1-1* and NPC1-overexpressing seedlings cultivated under control conditions

Figure S5. Molecular species composition of PC, PA, PE, PI and PG extracted from seven-day-old *Arabidopsis thaliana*

Figure S6. Molecular species composition of PC, PA, PE, PI and PG extracted from seven-day-old *Arabidopsis thaliana* after heat stress

Figure S7. Principal component analysis of PCs extracted from plants submitted or not to heat stress

Figure S8. Effect of HS on endogenous hormone levels in WT plants

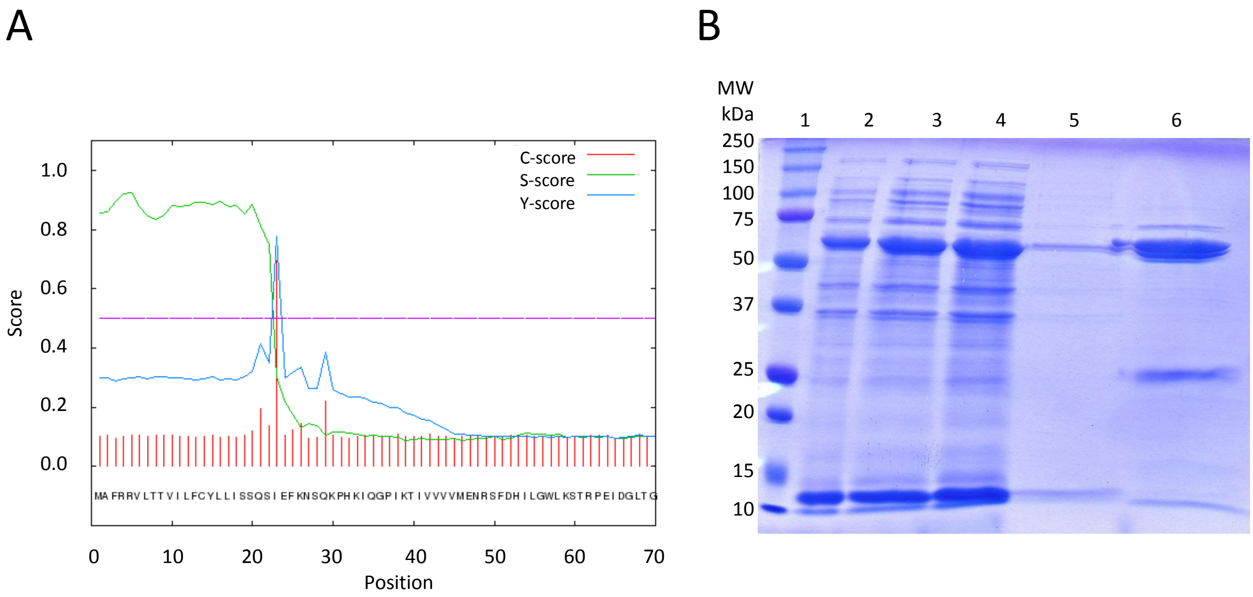

Fig. S1 Prediction of a signal peptide cleavage site and analyses of purification of the recombinant NPC1.* *(A) *The cleavage site between the signal peptide and coding region was identified between amino acids 22 and 23 using SignalP 4.1. .**(B)** Protein in each purification step was analyzed on a 12 % SDS-PAGE gel followed by Comassie blue staining. 1, marker; 2, culture supernatant; 3, flow through fraction; 4, 5 washing; 6, eluate from agarose with 250 mM imidazole.*

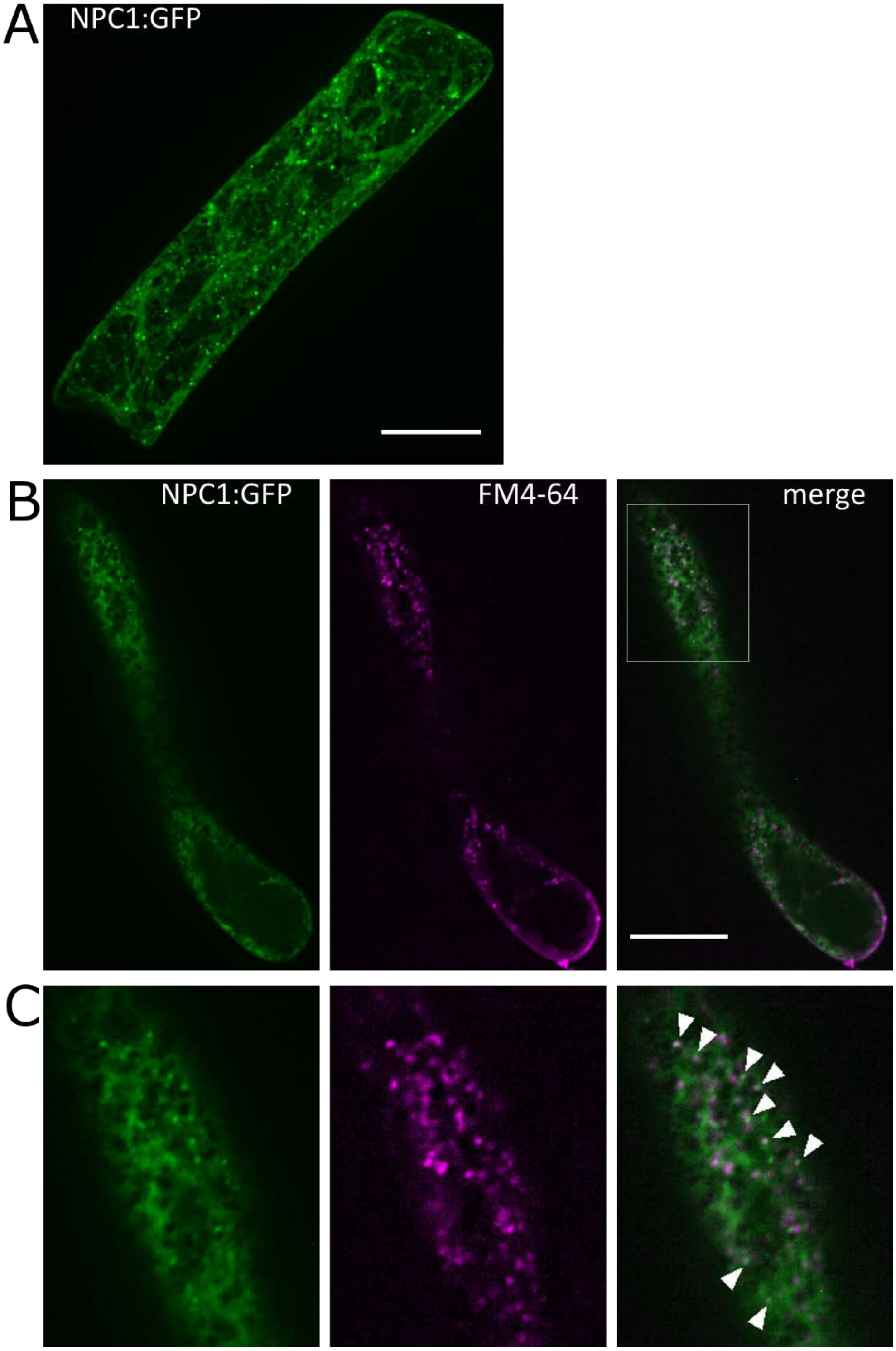

Fig. S2 NPC1:GFP localization in tobacco BY-2 cells. *BY-2 cells were transiently transformed by particle bombardment. After approximately 8 h of expression, cells were observed with a Nikon spinning disc confocal microscope.* ***(A)*** *NPC1:GFP is localized to the secretory pathway compartments in BY-2 cells. Maximum intensity projection was obtained from 88 slices containing Z-stack (Z-step 0.3 um) spanning from the cortical cytoplasm to the middle plane of the cell. Clear reticular signal was accompanied by many puncta.* ***(B)*** *Only a rather small percentage of NPC1:GFP puncta co-localized with FM4-64 labelled endomembranes. Transformed BY-2 cells were labelled with 5 µM FM4-64 solution on ice and then incubated for 15 min at room temperature to induce endocytosis.* ***(C)*** *A close-up of the rectangular region marked in (B). Merging reveals the co-localization of FM4-64 labelled endomembranes and NPC1:GFP puncta (arrowheads). Bars represent 20 µm.*

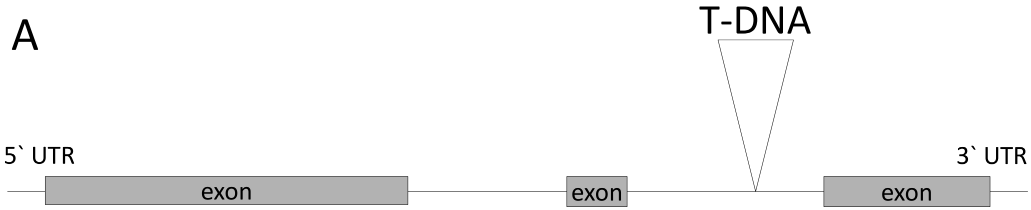

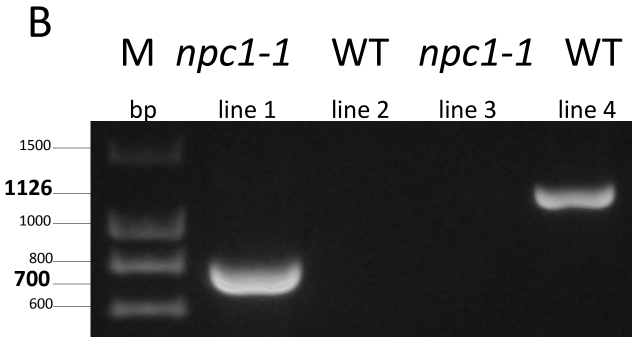

C

WT	1
npc1-1	0.04
NPC1-OE	111.3

Fig. S3 Characterisation of npc1-1 and NPC1-OE mutant lines. (A) *Position of the T-DNA insertion in the NPC1 gene.* ***(B)*** *PCR analysis of npc1. DNA was isolated from WT and npc1 plants. PCR was performed with a primer aligning to the T-DNA border on the left and a genomic primer on the right (lines 1, 2) and with left and right genomic primers (lines 3, 4).* ***(C)*** *Analysis of NPC1 expression in npc1-1 and NPC1-OE plants. RNA was isolated from WT, npc1 and 35S::NPC1 seedlings and quantitative RT-PCR was performed. SAND (At2g28390) was used as a reference gene.*

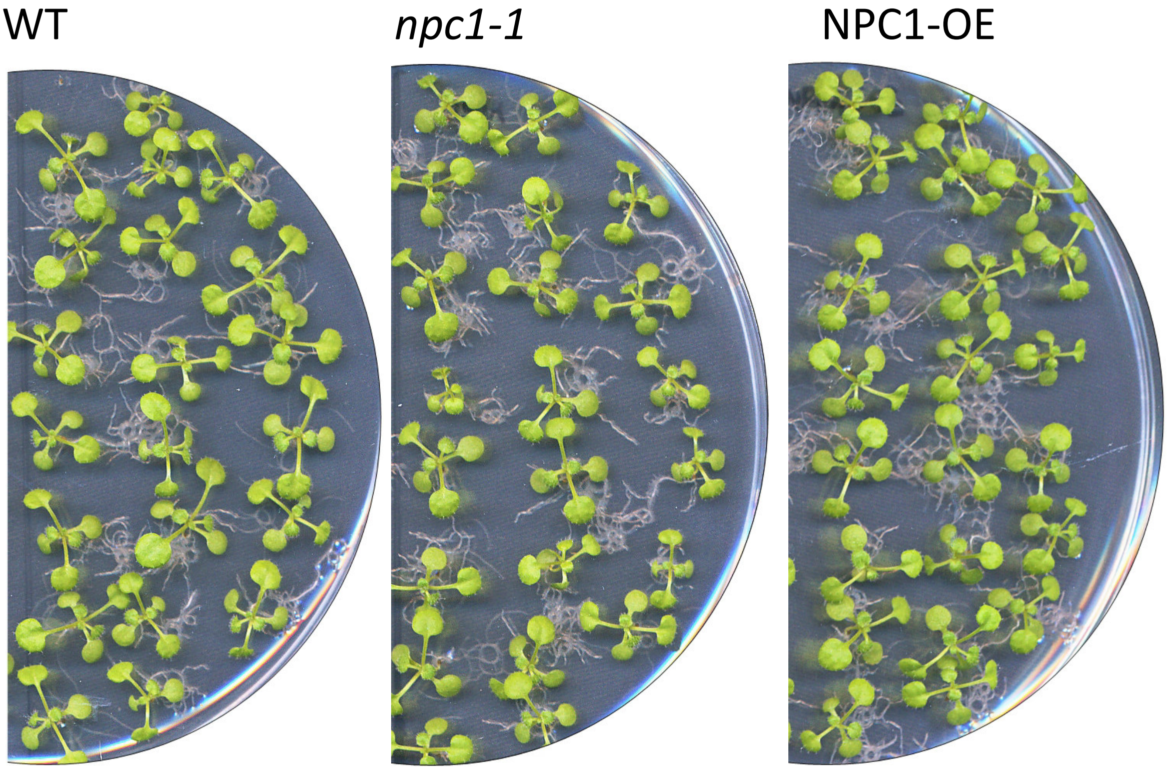

Fig. S4 Survival rate of Arabidopsis thaliana WT, npc1-1 and NPC1-overexpressing seedlings cultivated under control conditions. *Fourteen-day-old Arabidopsis seedlings were grown on agar plates at 22 °C (22 seedlings of each genotype on one plate) and the survival rate was determined. There were no variations between WT, npc1-1 and NPC1-OE. This experiment was repeated twice with similar results.*

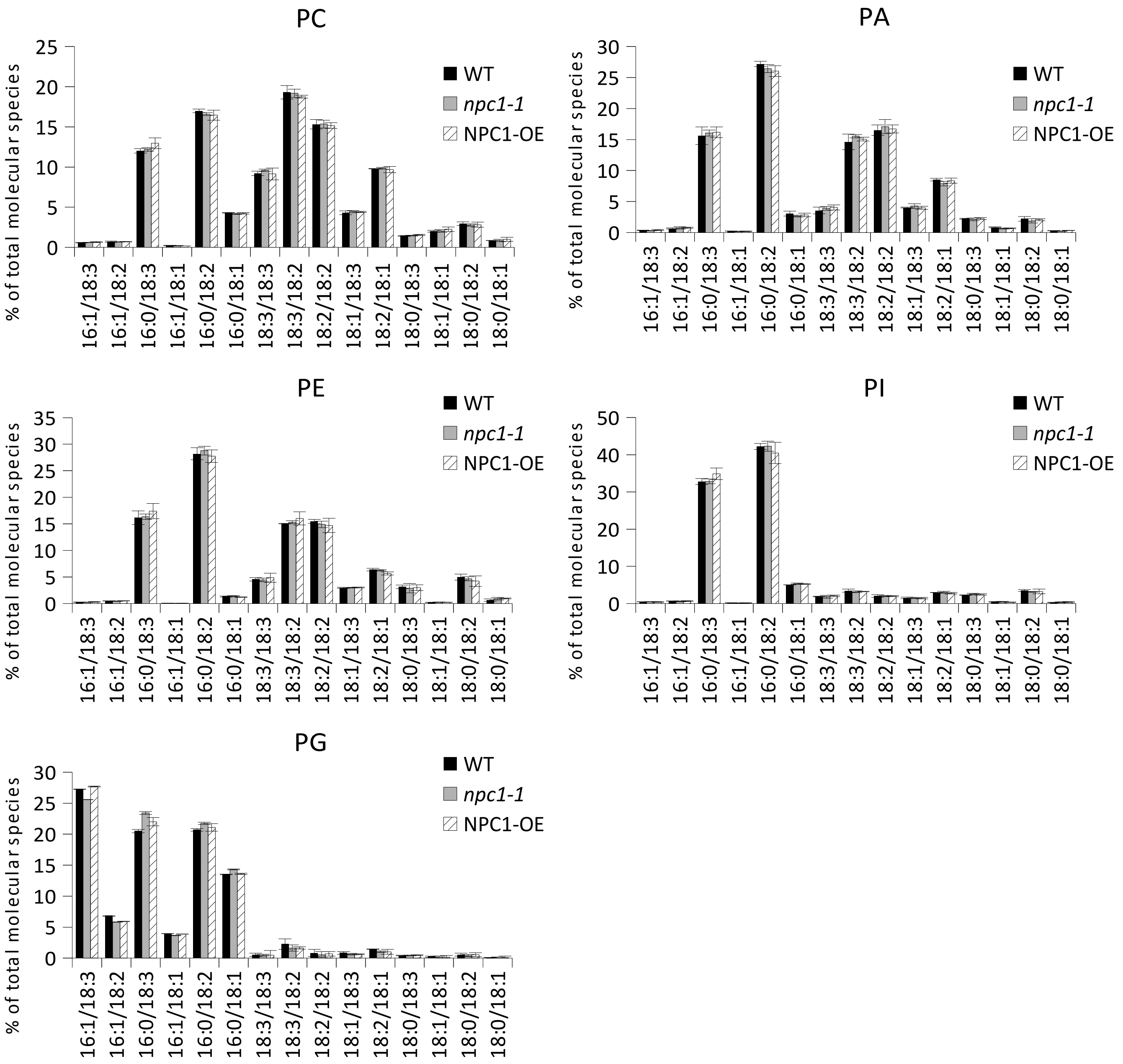

Fig. S5 Molecular species composition of PC, PA, PE, PI and PG extracted from seven-day-old Arabidopsis thaliana. *Arabidopsis seedlings (WT, npc1-1 and NPC1-OE) were grown on agar plates at 22 °C for seven days. Lipid analysis was performed in non-stressed plants. Values are means ±SD; one experiment includes 45 seedlings. WT, wild type; PC, phosphatidylcholine; PA, phosphatidic acid; PE, phosphatidylethanolamine; PI, phosphatidylinositol; PG, phosphatidylglycerol.*

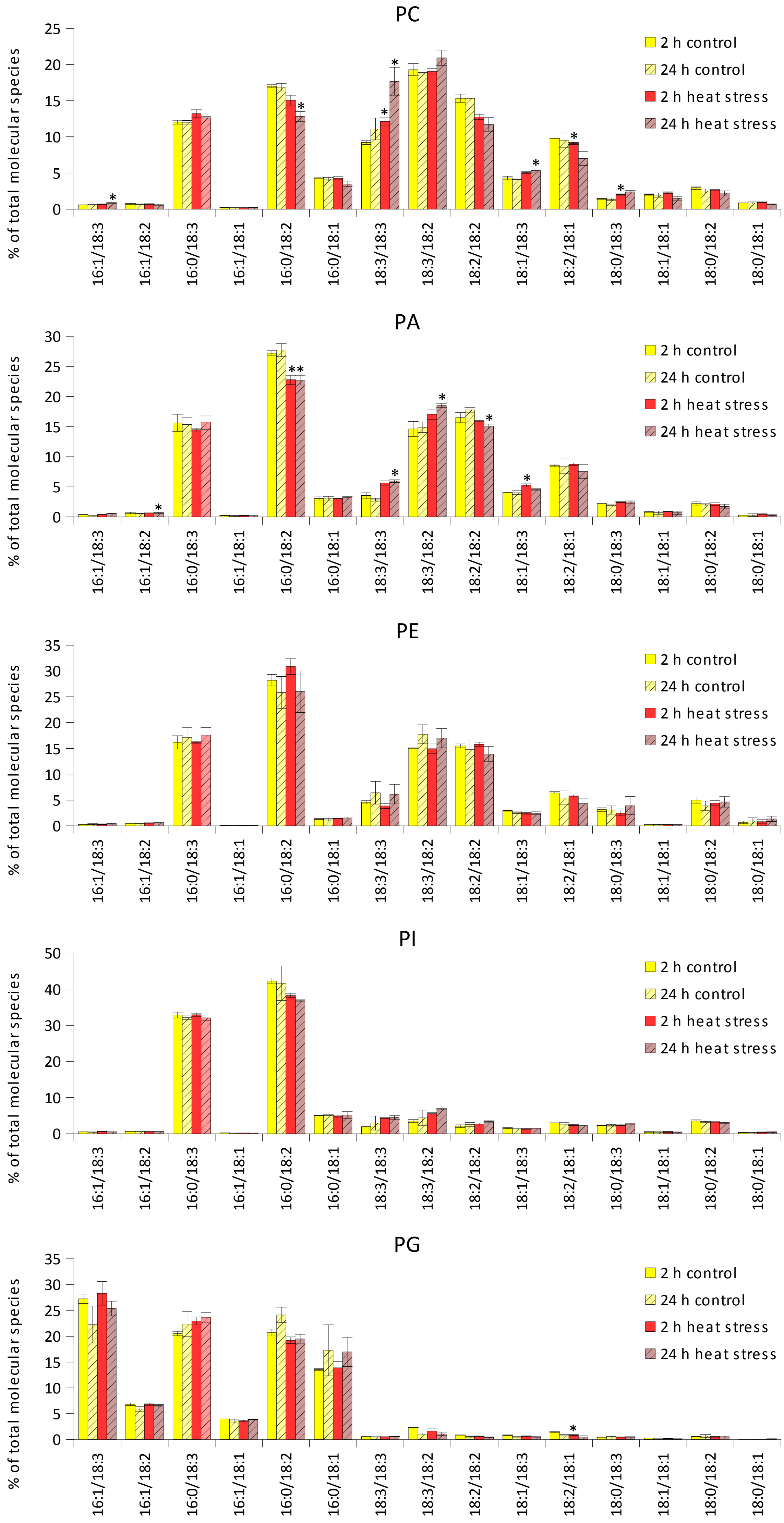

***Fig. S6 Molecular species composition of PC, PA, PE, PI and PG extracted from seven-day-old Arabidopsis thaliana after heat stress.** Arabidopsis WT seedlings were grown on agar plates at 22 °C for seven days. Lipid analysis was performed under control conditions and at 2 or 24 h after HS (45 min in 42 °C). Values are means ±SD; one experiment includes 45 seedlings. Statistical analysis indicates significant differences (*, P<0.05) between controls and heat stress using two-tailed Student`s test (n = 3). PC, phosphatidylcholine; PA, phosphatidic acid; PE, phosphatidylethanolamine; PI, phosphatidylinositol; PG, phosphatidylglycerol.*

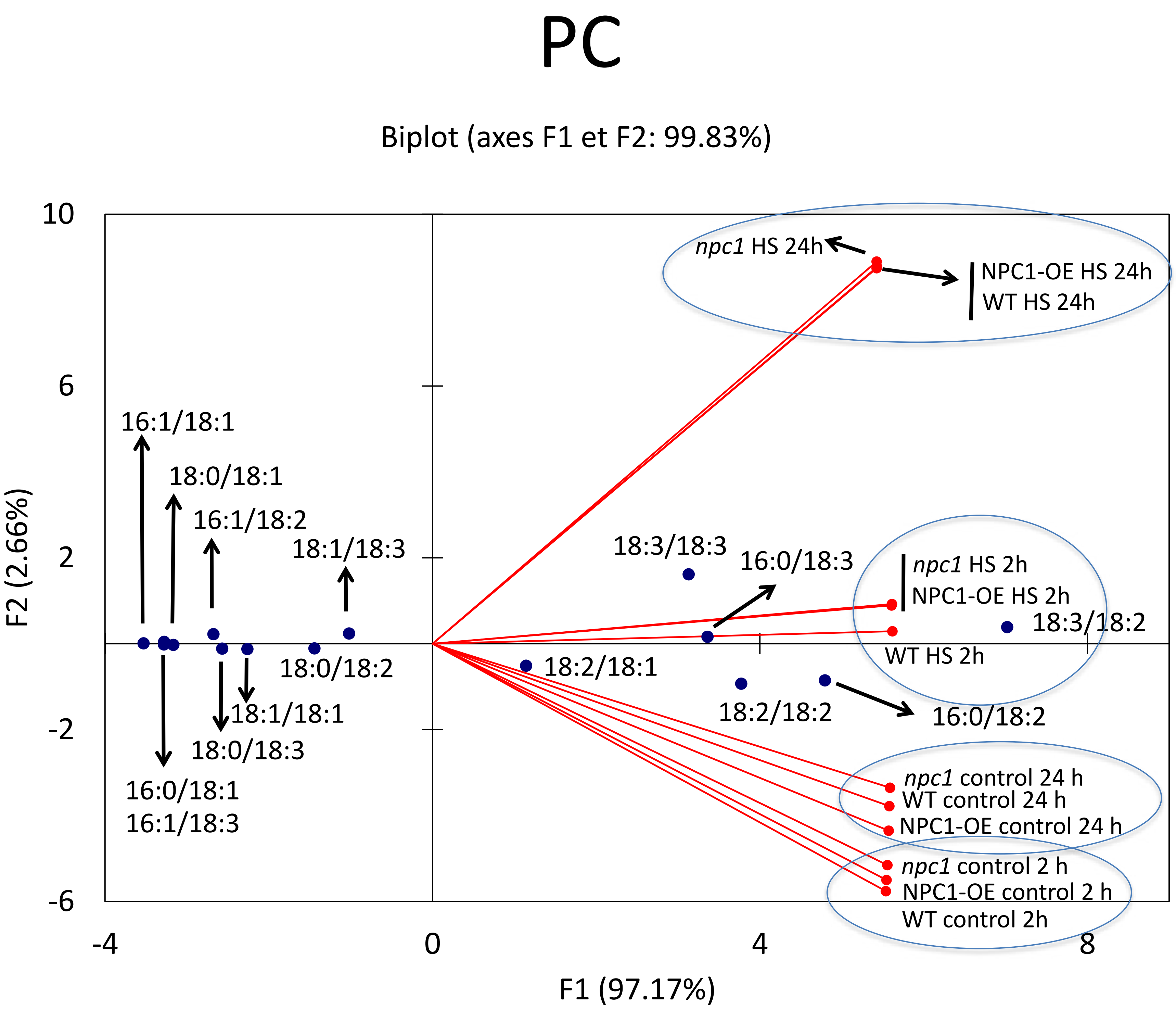

Fig. S7 Principal component analysis of PCs extracted from plants submitted or not to heat stress. *HS 24 h, PCs extracted in plants submitted to 45 min heat stress and back to control temperature for 24 hr; HS 2 h, PCs extracted in plants submitted to 45 min heat stress and back to control temperature for 2 h; control 24 h, PCs extracted in control plants at the same time as HS 24 h; control 2 h, PCs extracted in control plants at the same time as HS 2 h. PCA was calculated using Pearson's correlation matrix.*

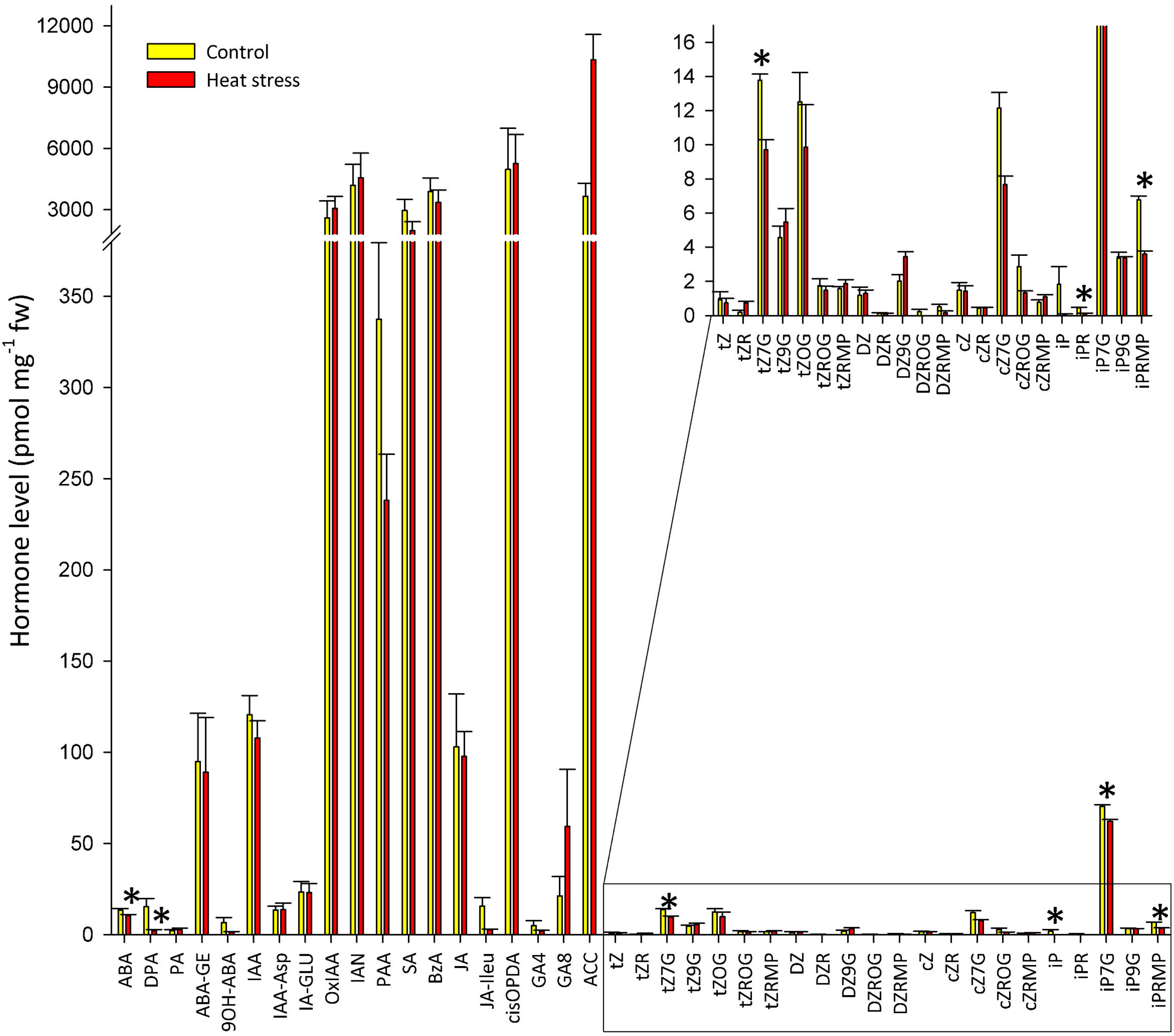

Fig. S8 Effect of HS on endogenous hormone levels in WT plants. *Seven day-old Arabidopsis seedlings were exposed to 42 °C for 45 min. Endogenous hormone levels were determined using high-performance liquid chromatography. Values are means ±SE; one experiment includes 124 seedlings. Statistical analysis indicates significant differences (*, P<0.05) between controls and heat stress using two-tailed Student`s test (n = 6). ABA, abscisic acid; DPA, dihydrophaseic acid; PA, phaseic acid; ABA-GE, ABA-glucose ester; 9OH-ABA, 9-hydroxy-ABA; IAA, indole-3-acetic acid; IAA-Asp, IAA-aspartate; IAA-Glu, IAA-glutamate; OxIAA, oxo-IAA; IAN, Indole-3-acetonitrile; PAA, phenylacetic acid; SA, salicylic acid; BzA, benzoic acid; JA, jasmonic acid; JA-Ile, JA-isoleucine; cisOPDA, cis-(+)-12-oxo-phytodienoic acid; GAn, gibberellin n = 4, 8; ACC, 1-aminocyclopropane-1-carboxylic acid; tZ, trans-zeatin; tZR, trans-zeatin riboside; tZ7G, trans-zeatin-7-glucoside; tZ9G, trans-zeatin-9-glucosid; cZOG, cis-zeatin-O-glucoside; tZROG, trans-zeatin riboside -O-glucoside; tZRMP, trans-zeatin riboside monophosphate; DZ, dihydrozeatin; DZR, dihydrozeatin riboside; DZ9G, dihydrozeatin-9-glucoside; DZROG, dihydrozeatin riboside -O-glucoside; DZRMP, dihydrozeatin riboside monophosphate; cZ, cis-zeatin; cZR, cis-zeatin riboside; cZ7G, cis-zeatin-7-glucoside; cZROG, cis-zeatin riboside -O-glucoside; cZRMP, cis-zeatin riboside monophosphate; iP, isopentenyl adenine; iPR, isopentenyl adenosine; iP7G, isopentenyl adenine-7-glucoside; iP9G, isopentenyl adenine-9-glucoside; iPRMP, isopentenyl adenosine monophosphate; fw, fresh weight.*
